# Amelioration of moisture stress effect by CaCl_2_ pre-treatment in upland rice

**DOI:** 10.1007/s40502-014-0058-y

**Published:** 2014-02-12

**Authors:** S. Helena Devi, M. Kar

**Affiliations:** Department of Plant Physiology, College of Agriculture, Orissa University of Agriculture and Technology, Bhubaneswar, 751003 Odisha India

**Keywords:** Rice, Moisture stress, Photosynthesis, CaCl_2_ treatment

## Abstract

Five varieties of rice viz., Subhadra, Ghanteswari, Sidhant, Jogesh and Khandagiri were grown to study the stress alleviating effect of CaCl_2_ at panicle initiation (PI) stage. CaCl_2_ (1 %) was given as seed treatment, which ameliorated the adverse effect of drought stress on photosynthesis (Pn), stomatal conductance (Gs), membrane stability index (MSI), leaf moisture retention index (LMRI) and grain weight. Under stress condition Pn, Gs, MSI and LMRI were significantly reduced. All CaCl_2_ pre-treatments enhanced yield, and the reduction under moisture stress was only 21.9 % as compared to 62 % in untreated stressed plants. Amongst the varieties Shubadra and Ghanteswari possessed better drought adaptive mechanism than Sidhant, Jogesh and Khandagiri.

Rice (*Oryza sativa* L.) is the predominant food crops of the world and staple food of more than 60 % of the global population, hence deserves top priority in agriculture system. However, most of the rice growing regions in India suffer from low productivity, mainly due to its cultivation in rainfed situation. In Odhisa rice is synonymous with food and the state was a leading rice producer in the country in the 1950s. But situation was reversed in the post-HYV period as Odhisa’s share in country’s rice production, which was more than 10 % in the pre-HYV period came down to 7 % in 1992–93 because of unprecedented natural calamities and frequent prevalence of biotic and abiotic stresses.

Rice is sensitive to moisture stress owing to its requirement of wetland ecosystem. Moisture stress frequently occurs, either at one or more phenological stage of upland rice crop rose under rainfed condition. Therefore, it is imperative to evaluate the performance of rice cultivar under moisture deficit condition (Majeed et al. 2010). Various morphological and physiological traits have been reported to be associated with drought resistance in plants growing naturally in arid environment. Identification of these traits is necessary to incorporate such desirable traits in the crop breeding programme (Baruah et al. 1998). Though attempts have been made to study how the plants overcome the impact of stress (on growth and yield reduction) on account of moisture deficit, a lot is yet to be understood as to the physiological and biochemical basis of drought tolerance in rice. This study has been taken up with the objective to have an insight into the physiological and biochemical basis of drought tolerance in rice, which would help in designing the crop ideotypes for drought prone environments.

The pot culture experiment was conducted in the wire-netting house of the Department of Plant Physiology, College of Agriculture, OUAT, Bhubaneswar. Five varieties of rice, viz., Subhadra, Ghanteswari, Sidhant, Jogesh and Khandagiri were taken for study. Required quantities of breeder seeds were collected from the EB-1, Department of Plant Breeding and Genetics, OUAT farm for the purpose. The experiment was the conducted in a factorial CRD with three replications and five cultivars of paddy with one level of water stress at panicle initiation (PI) stage and stress with 1 % CaCl_2_ treatment (foliar spray before commencement of drought stress/withholding water).

The rate of photosynthesis was measured on leaves using a portable Infrared Gas Analyzer (IRGA) (CIRAS-2 of version 2.02). Assimilation by the enclosed leaf area caused a depletion of CO_2_ concentration inside the chamber and this depletion was measured for three consecutive times of 10 s each. Based on the CO_2_ depletion and other factors such as temperature, pressure and volume of the enclosure, the rate of photosynthesis was computed by the inbuilt microcomputer of the IRGA, which was expressed in μmol m^−2 ^s^−1^. Stomatal conductance was measured using IRGA. The stomatal conductance was expressed as μmol m^−2 ^s^−1^.

Membrane stability index (MSI) was estimated according to Sairam et al. 2000. Two leaf samples were immediately kept in moist polythene bags. 100 mg of fresh leaf was taken and were cut into small pieces. One set kept in 10 ml distilled water was heated at 40 °C for 30 min and conductivity was measured in conductivity meter (C_1_) of ELICO-make. Another set in 10 ml distilled water was boiled at 100 °C for 15 min and conductivity was measured in conductivity meter (C_2_).

$$ {\text{MSI}} = \left( {1 - {\text{ C}}_{ 1} / {\text{C}}_{ 2} } \right) \times 100 $$

where, C1 & C2 are conductivity at 40 and 100 °C.

Leaf moisture retention index (LMRI) was determined as per Gupta and Sharma (Gupta and Sharma 2003). Fresh weight (FW) of leaf samples were taken and kept in laboratory at room temperature for 5 h. The ambient weight (AM) was taken and the samples was oven dried for 48 h at 60–65 °C in hot air oven.

$$ {\text{LMRI}} = \left[ {\left( {{\text{AM}} - {\text{DW}}} \right) /\left( {{\text{FW}} - {\text{DW}}} \right)} \right] \times 100 $$

where AM is ambient weight and DW is dry weight.

Yield components such as number of grains per panicle, grain yield, 1,000 grain weight and harvest index were recorded at maturity.

The net photosynthetic rate (Pn) and stomatal conductance (Gs) decreased in all cultivars under drought stress at PI stage but pre-treatment with CaCl_2_ (1 %) reduced the effect of drought stress (Table 1). A close association between leaf water and the rate of photosynthesis has been explained on the basis of partial or complete stomatal closure, which often resulted in lower internal CO_2_ concentration and limits photosynthesis through lowering ribulose-1,5-bisphosphate carboxylase/oxygenase (Rubisco) (Cornic et al. 1992). The photosynthetic rate decreased by 20.3 % under drought, while the reduction was 11.5 % in plant pretreated with CaCl_2_ as compared to control. The maximum photosynthesis rate was recorded in Subhadra followed by Ghanteswari and least was recorded in Sidhant. Stomatal conductance showed a reduction of 23.5 % under stress, whereas CaCl_2_ pre-treatment ameliorated the effect of stress on plants and increased the conductance by 52 %. Among the varieties highest Gs was observed in Subhadra followed by Khandagiri and Ghanteswari and lowest stomatal conductance was observed in Jogesh and Sidhant. The minimum reduction in stomatal conductance was recorded from Khandagiri (11.11 %) followed by Subhadra (23.3 %), while in stressed plants pre-treated with CaCl_2_ Gs increased by 4.5 in Khandagiri. Stomatal closure reduces water loss, but also reduces the gas exchange between the plant and the ambient air. The reduced CO_2_ intake then results in reduced photosynthesis (Chaves et al. 2002). This mechanism is therefore useful to improve plant survival under drought stress, but is also associated with yield reduction (Price 2002).

**Table 1:** Effect of drought stress on photosynthetic rate (Pn) (μmol m^−2 ^s^−1^) and stomatal conductance (Gs) (mmol m^−2 ^s^−1^) of different rice varieties at PI stage

Varieties	Photosynthesis rate (μmol m^−2 ^S^−1^)				Stomatal conductance (mmol m^−2 ^S^−1^)			
	Control	Drought stress	Drought stress + CaCl_2_	Mean	Control	Drought stress	Drought stress + CaCl_2_	Mean
V1	12.67	9.86	10.9	11.14	0.09	0.08	0.5	0.22
V2	12.37	12.87	11.27	12.17	0.17	0.09	0.13	0.13
V3	9.09	4.91	7.55	7.18	0.14	0.1	0.15	0.13
V4	17.67	12.97	14.7	15.11	0.21	0.16	0.27	0.21
V5	17.63	12.93	17	15.89	0.26	0.19	0.24	0.23
Mean	13.89	10.71	12.3	12.29	0.17	0.13	0.26	0.19
	V	S	VXS		V	S	VXS	
Se(m)	0.38	0.3	0.66		0.03	0.03	0.06	
CD 5 %	1.11	0.86	1.92		0.09	0.07	0.16	
CV (%)	9.34				51.18			

Leaf moisture retention index and membrane stability index are easily measurable physiological traits, which reflect the leaf turgor maintenance under stress (Table 2). Both these parameters decreased in all the cultivars with the imposition of moisture stress. In general, the reduction for LMRI and MSI was 40.9 and 51.0 %, respectively under stress and 23.7 and 19.8 % with CaCl_2_ pre-treatment as compared to control. Among the cultivars Shubadra maintained high LMRI (11.1 %) and MSI (52.67 %). The rate at which an excised leaf loses water has been related to its ability to lose under moisture stress condition in the field. Higher leaf water retention index under water stress appears to be associated with drought tolerance, resulting in better survival under water stress condition. This trait under field condition was observed to be associated with higher seed/grain yield (Laxmi et al. 2009).

**Table 2:** Effect of drought stress on leaf moisture retention index (LMRI) and membrane stability index (MSI) in leaves of different rice varieties at PI stage

Varieties	LMRI (%)				MSI (%)			
	Control	Drought stress	Drought stress + CaCl_2_	Mean	Control	Drought stress	Drought stress + CaCl_2_	Mean
V1	10.46	5.73	7.83	8	52.67	26.27	46	41.64
V2	12.54	7.98	9.88	10.14	54.93	21.63	44.5	40.36
V3	12.42	8.25	9.66	10.11	71.9	37.07	52.83	53.93
V4	14.37	8.15	10.17	10.89	57.33	31.6	49.4	46.18
V5	14.25	7.73	11.31	11.1	65.7	31.53	52.67	46.97
Mean	12.81	7.57	9.77	10.05	60.55	29.62	49.08	46.42
	V	S	VXS		V	S	VXS	
Se(m)	0.52	0.4	0.9		1.06	0.82	1.84	
CD 5 %	1.5	1.17	2.61		3.08	2.38	5.33	
CV (%)	15.5				6.87			

Reduction in the number of panicle by water stress was 8.3 % and CaCl_2_ pre-treatment reduced the adverse effect of stress on panicle number to 4.8 % as compared to control (Table 3). The number of grain per panicle was significantly reduced with concomitant increase in chaffs, which also corroborated with the findings of Babu et al. (2003). The application of ameliorants increased the number of grains per panicle, which is an important trait for higher productivity (Gupta and Sharma 2003). Drought at initiation of reproductive stage hampers spikelet number, increased spikelet sterility and lowered seed yield (Das and Kar 2005). Spikelet fertility was reduced by 13 % when rice plants were exposed to water stress (Babu et al. 2003). Water stress at PI stage resulted in a reduction in grain yield, harvest index and 1,000 grain weigh by 21.9, 22.7 and 29.7 % and 6.2, 3.18 and 12.6 % in CaCl_2_ pre-treated stressed plants, respectively as compared to control (Table 3). As photosynthesis becomes inhibited by drought the grain filling process becomes increasingly reliant on stem reserve utilization. Stem reserve mobilization capacity is related to yield under water-stress condition (Blum 2000). The positive relation obtained between yield and harvest index is indicative of better dry matter partitioning into economically useful parts (Mishra et al. 2000). The highest grain yield was obtained in Subhadra (32.28 g/hill), followed by Khandagiri (31.53 g/hill), while lowest was obtained in Jogesh (26.39 g/hill). The production and retention of fertile spikelet, productive tillers and grain per panicle were found to be positively co-related to rice productivity under stress. Application of CaCl_2_ (1 %) as seed treatment helped in mitigating the harmful effect of drought stress. In conclusion it can be suggested that among the tested varieties Subhadra and Ghanteswari were more efficient as it possess a better drought tolerance system. Nevertheless, further investigation under varying field conditions will add more insight to this aspect, which will provide a strong impetus for the researchers/scientists to explore the possibilities in crop improvement programme.

**Table 3:** Effect of drought stress on yield attributes of different varieties of rice plant at PI stage

Varieties	Grain weight (g panicle^−1^)				Grain weight (g plant^−1^)				1,000 grain weight (g)				Harvest index (%)			
	Control	Drought stress	Drought stress + CaCl_2_	Mean	Control	Drought Stress	Drought stress + CaCl_2_	Mean	Control	Drought stress	Drought stress + CaCl_2_	Mean	Control	Drought stress	Drought stress + treatment	Mean
Khandagiri	0.98	0.76	0.91	0.89	7.02	5.45	6.41	6.29	18	11	16.33	15.11	41.5	33.23	38.17	37.63
Jogesh	0.95	0.76	0.85	0.85	6.93	5.36	6	6.1	18.33	13.67	18.33	16.78	35.9	29.3	31.2	32.13
Sidhant	0.97	0.76	0.86	0.86	7.03	5.35	5.84	6.07	30.33	22	27	26.44	44.47	35.03	40.3	39.93
Ghanteswari	1.02	0.76	0.92	0.9	6.87	5.28	6.1	6.08	36	26	29	30.33	46.03	33.93	57.57	45.84
Subhadra	1.02	0.81	0.95	0.93	7.28	5.61	6.47	6.45	23.8	16.73	20.8	20.44	41.45	32.05	40.13	37.88
Mean	0.99	0.77	0.9	–	7.03	5.41	6.16	–	25.29	17.88	22.29	–	41.87	32.71	41.47	–
	V	S	VXS		V	S	VXS		V	S	VXS		V	S	VXS	
Se(m)	0.017	0.013	0.025		0.121	0.093	0.29		0.5	0.39	0.87		2.55	1.98	4.42	
CD 5 %	0.049	0.038	0.079		0.349	0.27	0.605		1.46	1.13	2.52		7.38	5.72	12.79	
